# Functional Role and Diagnostic Potential of Biomarkers in the Early Detection of Mastitis in Dairy Cows

**DOI:** 10.3390/ani16020159

**Published:** 2026-01-06

**Authors:** Eleonora Dall’Olio, Melania Andrani, Mario Baratta, Fabio De Rensis, Roberta Saleri

**Affiliations:** 1 Department of Veterinary Science, University of Parma, 43126 Parma, Italyfabio.derensis@unipr.it (F.D.R.); roberta.saleri@unipr.it (R.S.); 2 Department of Chemistry, Life Sciences and Environmental Sustainability, University of Parma, Parco Area delle Scienze 11a, 43124 Parma, Italy; mario.baratta@unipr.it

**Keywords:** mastitis, dairy cows, biomarkers, diagnosis, microRNA

## Abstract

Mastitis remains a prevalent and economically detrimental disease within the dairy industry, profoundly affecting animal welfare, milk quality, and overall production output. Nowadays, Somatic Cell Count (SCC) is widely recognized as the gold-standard indicator for the detection of mastitis; however, its limitations in pathogens discrimination and the lack of early-stage characterization of mastitis highlight the need for complementary diagnostic approaches. This review synthesizes recent research into the development and validation of novel biomarkers for the early and accurate identification of mastitis in dairy cows. The investigation encompasses a range of biological molecules for improving mastitis diagnosis. Biomarkers such as lactoferrin (LTF), β-defensin 4 (DEFB4), vitronectin, paraoxonase 1 (PON1), and N-acetyl-β-D-glucosaminidase (NAGase) show promise in distinguishing between cows not susceptible and cows susceptible to mastitis. Concurrently, nucleic acid-based biomarkers are emerging as a particularly promising frontier. While mitochondrial DNA (mtDNA) has demonstrated insufficient specificity, microRNAs (miRNAs) are gaining attention as highly stable and sensitive indicators of intramammary inflammation, potentially enabling the detection of subclinical infections before they become clinically apparent. Despite these advances, significant challenges related to specificity, reliability, and cost-effectiveness currently hinder the widespread practical application of any single biomarker. Therefore, future research should be directed towards the validation of a synergistic panel of multiple biomarkers to improve mastitis management in dairy cow farms.

## Simple Summary

Mastitis is one of the most common and costly diseases in dairy farming, with major impacts on cow welfare, milk quality, and farm productivity. Nowadays, somatic cell count can be still considered the gold standard indicator for diagnosis but lacks sensitivity for early or subclinical mastitis and cannot identify the pathogens involved. Therefore, recent study has been developed to identify biomarkers that could reveal subclinical mastitis and/or identify the pathogens involved. Several promising molecules, including lactoferrin, β-defensin 4, vitronectin, paraoxonase 1, N-acetyl-β-D-glucosaminidase, and microRNAs, have shown potential in detecting intramammary inflammation, with microRNAs emerging as particularly stable and sensitive indicators. Although these advances are encouraging, no single biomarker has yet to be accurate and reliable enough for routine use on farms, and future diagnostic strategies will likely rely on combined biomarker panels to improve early detection and mastitis management.

## 1. Introduction

Recent years have seen an increase in production of bovine milk and dairy products through intensive production systems. This has had some negative feedback regarding the health and welfare of dairy cows, all linked to a greater incidence of metabolic diseases, such as laminitis or ketosis, but especially mastitis [1], which is the only dairy cow disease capable of causing a lowering in milk value [2].

Mastitis in cows is an inflammation of the mammary gland (udder) usually caused by bacterial infection, leading to pain, swelling, heat, and redness, resulting in abnormal milk (clots, blood), and it can be clinical (visible signs) or subclinical [3,4]. Mastitis is one of the main problems for the milk industry because it reduces milk production, and milk produced by affected animals is of poor quality (high somatic cell number) and therefore cannot be consumed or sold. Bovine mastitis is associated with a daily loss of milk ranging from 1.0 to 2.5 kg per animal in the first two weeks after onset and with a total loss ranging from 110 to 552 kg during the entire lactation period [5]. Costs associated with the treatment or premature culling of the affected cows must be added to these losses. Indeed, 49% of the financial losses caused by mastitis are due to reduced milk production and higher waste due to low quality, and 37% to culling and treatment costs [5]. It has been reported that, on average, the total failure cost due to bovine mastitis has been estimated to be USD 147 per cow per year, particularly attributed to milk production losses and culling, which represents 11% to 18% of the gross margin per cow per year [6], and that mammary tissue damage leading to decreased milk production accounts for 70% of the total losses [7].

The USA National Mastitis Council has developed the “5-Point Plan” program for the control of this disease: post-milking teat dipping; use of dry cow therapy at the end of each lactation; treatment of clinical cases; culling of cows presenting the disease in a chronic form; effective maintenance of milk equipment to ensure vacuum at the end of the teat [8].

It has also been highlighted that a reduction in fertility and an increase in time to conception in affected cows, even in the case of mild inflammation, poses a further problem that is both clinically and subclinically linked to mastitis, underlining the importance of research in this area [9,10].

For these reasons, the dairy industry is adopting strategies for the prevention of mastitis mainly based on increasingly stringent sanitary standards, such as the previously mentioned “5-Point Plan”, to significantly reduce the use of antibiotics and losses due to low milk quality and treatment costs. Indeed, as Murphy [11] stated, the thought of being able to control mastitis only using antibiotic treatments “is merely cutting the tops off the weeds and leaving the roots”. For example, the adoption of a preventive strategy based on selective antibiotic dry cow therapy (aDCT) at the end of the lactation period is increasing compared to the classic aDCT. Selective aDCT provides treatment only of infected or presumed-infected cows, but not of all dairy cattle [12,13]. Other attempts have seen the development of methods of genetic selection for mastitis resistance or immunization of cows using vaccines [14,15,16,17,18,19,20,21], but in both cases, there are limitations due to the vast range of pathogens capable of causing this infection.

In this context, improving the early detection and accurate diagnosis of mastitis represents a key factor in the dairy industry, highlighting the need for innovative and reliable biomarkers capable of supporting effective prevention and management strategies. The aim of this review has been to describe the biomarkers that have been identified or proposed with the characteristic of being able to provide an early diagnosis of mastitis in dairy cows. The effectiveness of each biomarker will be discussed and their combination for practical application in farms will be suggested. The literature considered in this review was selected through a targeted search of studies published in English between 2000 and 2025, retrieved from the PubMed database, and included both experimental and field studies addressing diagnostic approaches and biomarkers for clinical and subclinical bovine mastitis.

## 2. The Mastitis Classification

Risk factors, therapeutic strategies, and treatment for mastitis have been recently well reviewed [5,22].

Basically, mastitis arises when an inflammatory process develops in the mammary gland, with its main cause being infections by pathogenic bacteria, among which the prevalent ones are *Staphylococcus aureus* (*S. aureus*), environmental streptococci (*S. agalactiae*, *uberis* and *dysgalactiae*), coliform pathogens (*Escherichia coli* and *Klebsiella* spp.), and other Gram-negative bacteria [5,23,24]. These infections are classified into two categories based on epidemiology: contagious form when a healthy cow is infected by an affected one during milking, with the farmer’s hands or the milking machines as a vehicle; environmental form caused by ubiquitous bacteria that have nothing to do with the milking process but can be present in the farm environment, such as bedding, soil, and feces [5].

Mastitis can also be classified according to the form in which it occurs. We can therefore refer to clinical and subclinical mastitis. In case of clinical mastitis, typical signs of mammary gland inflammation (as described in the previous chapter) are evident, together with general clinical signs, such as fever, with temperatures above 39.5 °C, and loss of appetite [5]. The variability in the severity of the clinical signs and their greater or lesser evidence is closely linked to the type of pathogen causing the infection: Gram-negative bacteria such as *E. coli* cause a rapid and intense immune response; Gram-positive bacteria are responsible for slower and more moderate immune responses; *S. aureus* causes a very small and sometimes undetectable immune response [25,26,27].

Subclinical mastitis is more difficult to identify as it does not present visible clinical symptoms and can be identified thanks to changes in the composition of milk, as evidenced by somatic cell count (SCC) or microbiological analysis [4,5].

## 3. Current Diagnostic Methods for Bovine Mastitis

SCC is a well-established procedure that gives results in a short amount of time with lower costs, and it is still the gold standard for clinical mastitis diagnosis. It is a direct counting method under the microscope with the use of methylene blue staining [28]. Currently, milk samples subjected to SCC that reach the threshold of 2.0 × 10^5^ somatic cells/mL are considered indicative of subclinical mastitis [4,29].

However, SCC measurements are not always feasible as cow-side tests in the field [5,30] and can occasionally give false-positive or false-negative results [31], particularly in early subclinical or clinical mastitis, when the somatic cell number has not yet exceeded the threshold of 2.0 × 10^5^ somatic cells/mL [32]. It should also be noted that somatic cells include both immune cells and epithelial cells [33]; therefore, their total number can be influenced not only by infection or inflammation but also by physiological factors such as lactation stage and herd management practices [34].

There is also an indirect counting method (for a complete review of diagnostic method available to date see Chakraborty et al. [35]), the California mastitis test (CMT) described in 1957 [36], which has been recognized and accepted as a rapid and easy-to-perform test for determining SCC in milk quarter samples [37]. According to the method described by Schalm and Noorlander [36], CMT is performed by collecting 2 mL milk samples from each udder and using a paddle with four wells. An appropriate quantity of reagent composed of a 3% sodium lauryl sulfate and 1:10,000 bromocresol solution is added to each well; this way, the solution of milk and reagent will form thickenings proportional to the quantity of somatic cells contained in the samples but require a visual and subjective interpretation of the results [8]. However, both the SCC and the CMT present non-negligible problems. Although the CMT is the most widely used indirect test, this whole category of analysis has limitations related to a high percentage of false-positive or false-negative results [35]. However, because of the above limitation of the CMT, the SCC provides more reliable results but, unlike the CMT, it requires a more elaborate diagnostic test and more time to obtain the results.

Overall, research is increasingly oriented towards the evaluation of new biomarkers that give greater reliability in terms of the certainty of results and can have predictive characteristics to be able to identify the affected cows in very early stages of the disease. The following part of this review will provide an overview of the new biomarkers identified and proposed thus far that could serve as potential tools for the early detection and management of mastitis in dairy cows.

## 4. Characteristics of a Valid Biomarker

A biomarker is a biological marker, such as a DNA sequence or protein, intended for a correct clinical diagnosis. Based on their intrinsic characteristics, biomarkers can have multiple applications, and the same marker at different thresholds and conditions can be indicative of different endpoints. Among their functions, biomarkers can therefore be used to screen, diagnose, and monitor a given disease, as well as evaluate the response to a drug during therapy or even as prognostic indicators [38,39]. An effective biomarker should exhibit high specificity and sensitivity. Its concentration should therefore vary specifically in response to changes due to the conditions under investigation [38]. Considering these general characteristics, the next step is to examine the structural and functional classes of biomarkers that have been investigated specifically for mastitis, highlighting their potential for early detection and accurate diagnosis.

## 5. Structural and Functional Classes of Mastitis Biomarkers

Mastitis remains one of the most economically significant diseases in dairy industry, and the identification of reliable biomarkers for its early detection has become a key research focus. Several biological factors can be explored for the development of such biomarkers, capable of distinguishing both clinical and subclinical forms of the disease. Depending on their chemical structure and biological role, potential biomarkers can be broadly classified into protein, glucidic, and molecular biomarkers. Protein biomarkers include acute-phase proteins, enzymes, and milk-derived proteins that reflect inflammatory processes or tissue damage in the mammary gland [40]. Glucidic biomarkers, such as milk lactose, are indicators of alterations in mammary epithelial metabolism and permeability associated with infections [4]. Molecular biomarkers, particularly microRNAs (miRNAs), have emerged as promising molecular indicators due to their role in post-transcriptional gene regulation during host–pathogen interactions or inflammation [41,42,43].

Considering their diverse biological origins, integrating these different biomarker categories could enhance diagnostic sensitivity and specificity, paving the way for more accurate and non-invasive detection methods in dairy herd management. A summary of the potential biomarker discussed in this review, including their sample type, associated mastitis form, and typical expression trends, is provided in Table 1.

### 5.1. Protein Biomarkers for Mastitis

Protein biomarkers are based on the detection of antimicrobial, inflammatory, and immunomodulatory factors released in milk and blood during the onset and progression of udder inflammation. These molecules reflect different stages and mechanisms of the host response to infection. Because their expression and activity may change earlier and more specifically than traditional indicators such as SCC, protein biomarkers have gained increasing attention as potential tools for the early diagnosis, pathogen characterization, and monitoring of mastitis severity. The following section focuses on protein candidates recently proposed as emerging biomarkers for the diagnosis and characterization of mastitis. For each candidate marker, this review focuses on their biological roles, diagnostic potential, and the main advantages and limitations emerging from recent studies.

#### 5.1.1. Lactoferrin

Lactoferrin (LTF) is an iron-binding glycoprotein with antimicrobial activity present in milk, stored in neutrophil granulocytes, and belonging to the transferrin family [50]. Its antimicrobial activity mainly results from iron sequestration, limiting essential nutrients for pathogenic microorganism and supporting innate immune defense in the mammary gland [47].

LTF concentrations in milk vary with mammary gland health, parity, and lactation stage, with higher levels in mastitic cows [47,48,49,50,55,56]. LTF content is typically highest in primiparous cows (0.08 ± 0.01 mg/mL) and tends to decrease with successive lactations [50,55,56]. Moreover, milk collected in early lactation (≤120 days in milk) generally contains higher LTF concentrations than samples from middle and late stages of lactation [50]. Musayeva et al. [50,55] also investigated the relationship between LTF and immunoglobulin G (IgG) concentrations, finding a strong positive correlation (r = 0.73) between the two proteins in both healthy and inflamed quarters. This suggests that LTF and IgG may act synergically as part of the mammary gland local immune defense. Seasonal variation and the infecting pathogen further modulate LTF levels, with higher concentrations reported during spring and in milk from cows infected by minor mastitis pathogens, such as *Corynebacterium* spp. and coagulase-negative staphylococci [49,50]. 

Raj et al. [47] also observed increased LTF levels in milk from cows with subclinical mastitis, accompanied by elevated serum concentrations of three acute-phase proteins: haptoglobin (Hp), α-1-acid glycoprotein (AGP), and serum amyloid A (SAA). Among these, SAA and Hp were identified as the main acute-phase proteins associated with bovine mastitis, while AGP and milk amyloid A (MAA) were also linked to the inflammatory response in the mammary gland, showing a strong positive correlation with SCC, with very low concentrations in milk from healthy cows [51].

In conclusion, despite consistent evidence of increased LTF during mastitis, considerable variability exists in the reported concentrations among the studies (Table 2). Such discrepancies in absolute values may arise from differences in analytical methods, causative pathogens, and physiological status (parity, lactation stage), making it difficult to define universal diagnostic thresholds. Therefore, while LTF alone may not provide sufficient specificity for subclinical mastitis diagnosis, its combined assessment with other acute-phase and immune-related proteins (IgG, Hp, AGP and MAA) could enhance diagnostic accuracy by reflecting both local and systemic inflammatory responses.

#### 5.1.2. β-Defensin 4

Defensins are a family of antimicrobial and immunomodulatory proteins expressed constitutively or inducibly, subdivided into α-, β-, and θ- defensins according to their molecular structure.

Previous transcriptomic analyses identified the expression of the β-defensin 4 (DEFB4) gene in primary mammary epithelial cells (PMECs) in cows affected by *E. coli*-induced mastitis [57], and subsequent studies confirmed its expression also in mastitis induced by *Staphylococcus* spp. [58]. Based on these findings, Neumann et al. [44] investigated DEFB4 concentrations in serum and milk to investigate its potential as a biomarker for clinical and subclinical mastitis. The study revealed that DEFB4 concentrations varied significantly according to the severity of infection: the highest levels were observed in cows with acute mastitis, whereas the lowest were detected in those with subclinical mastitis. Compared with healthy controls (153 pg/mL in serum and 97 pg/mL in milk), DEFB4 levels were significantly elevated in acute mastitis cows serum (245 pg/mL) but markedly reduced both in serum (85 pg/mL) and milk (46 pg/mL) during subclinical infections. Interestingly, in milk, DEFB4 concentrations increased during the acute mastitis and became significantly different from controls only after 12 days (115 pg/mL at day 1 vs. 192 pg/mL at day 12), indicating that this peptide seems to dynamically reflect the progression of the inflammatory response. 

To summarize, Neumann et al. [44] reported that DEFB4 concentrations were increased during acute mastitis and were interestingly lower in both serum and milk from cows with subclinical mastitis compared to healthy controls. This counterintuitive finding may reflect an early consumption of or depletion of constitutively expressed defensins before the activation of an inducible immune response in the mammary gland. In contrast, during acute clinical mastitis, a marked systemic release of DEFB4 occurs, followed by a local increase in these protein levels within the milk, suggesting a biphasic response involving both systemic and local defense mechanisms. Such dynamic modulation suggests that DEFB4 expression is linked to disease severity and stage, rather than simply reflecting the presence of infection. Therefore, despite the need of further validation, DEFB4 can be considered a promising biomarker candidate for the early detection and differentiation of mastitis forms.

#### 5.1.3. Vitronectin

Vitronectin (or bovine S-protein) is a multifunctional glycoprotein involved in coagulation, cell invasion and adhesion, and modulation of inflammatory responses, including the recruitment of neutrophils [48]. It has also been implicated in pathogen adhesion to host cells, thus promoting infection and inflammation [59]. Increased serum concentration of vitronectin has been reported in both subclinical (22%) and clinical (37%) mastitis compared with healthy controls [48]. The consistent upregulation observed across disease forms suggests local production at the site of infection with subsequent release into circulation, in line with its immunomodulatory functions [48,59]. Although further validation is required, these findings support vitronectin as a potential serum biomarker for the early detection of subclinical mastitis.

#### 5.1.4. Paraoxonase 1

Paraoxonase 1 (PON1) is a liver-synthesized enzyme associated with high-density lipoproteins (HDLs) and plays a key role in antioxidant and anti-inflammatory defense mechanisms [45,48]. Turk et al. [48] reported a reduction in serum PON1 activity in cows with both clinical and subclinical mastitis, supporting the involvement of oxidative stress in mastitis pathogenesis. This decrease was more pronounced in clinical mastitis (237 ± 61 U/I) than in subclinical cases (268 ± 47 U/I) when compared with healthy controls (305 ± 63 U/I), suggesting a disease severity-dependent modulation of PON1 activity [48]. Further evidence was provided by Nedić et al. [45], who observed reduced PON1 activity in both serum and milk from cows affected by *S. aureus*-induced subclinical mastitis, with greater reductions associated with higher bacterial loads. Therefore, a negative correlation emerged between PON1 activity and SCC, indicating decreasing enzyme activity with increasing pathogen burden [45]. These studies therefore suggest PON1 as a potential biomarker for subclinical mastitis, especially thanks to the high sensitivity in the variation in its activity in the presence of this disease; however, the comparison with SCC does not provide better information.

#### 5.1.5. N-Acetyl-β-D-glucosaminidase

N-acetyl-β-D-glucosaminidase (NAGase) is a lysosomal glycosidase released into milk by neutrophils following mammary tissue damage, and it is commonly used as an indicator of inflammation in combination with SCC. NAGase increases during mastitis, with higher levels in infections caused by major pathogens (e.g., *E. coli*) compared with minor pathogens (e.g., *Corynebacterium*) [52,60,61,62]. Consistently, cows with clinical mastitis show higher NAGase concentrations (>28.0 pmol 4-MU/min/μL) than those with subclinical mastitis (11.5 pmol 4-MU/min/μL), reflecting the greater degree of tissue damage associated with clinical forms [61]. Physiological factors also influence NAGase activity. Hovinen et al. [52] reported increased values during the first 30 days in milk (DIM), while for DIM ≥ 30, a reference range of 0.1–1.04 pmol 4-MU/min/μL was proposed for healthy cows. Based on these findings, specific cut-off values were suggested to discriminate healthy milk from subclinical mastitis (0.76 pmol 4-MU/min/μL) and clinical mastitis (1.19 pmol 4-MU/min/μL), as well as to distinguish clinical from subclinical forms (5.3 pmol 4-MU/min/μL), with high sensitivity, specificity, and accuracy [52]. Overall, NAGase reflects inflammation severity and tissue damage and may support the differentiation between subclinical and clinical mastitis, particularly when interpreted alongside SCC and pathogen-related information. 

#### 5.1.6. Cathelicidins

Cathelicidins are antimicrobial and pro-inflammatory proteins found in neutrophil secondary granules, consisting of a cathelin domain and C-terminal portion. They are released during mammary gland inflammation, making their abundance in milk a sensitive indicator of both clinical and subclinical mastitis [53]. Milk concentrations of cathelicidins vary with mastitis severity: they are undetectable in healthy controls, higher in subclinical cases, and highest in clinical mastitis, reflecting the degree of neutrophil activation. Seven cathelicidin types have been identified in dairy cows, and their concentrations correlate positively with SCC [53]. Cathelicidin-1, in particular, has been proposed as a potential biomarker for subclinical mastitis, showing a significant correlation with mastitis severity [63]. Furthermore, Addis et al. [64] report that the microorganism causing the greatest increase in cathelicidins was *S. agalactiae*, while those responsible for the least increase were *Serratia* spp., according to their respective pathogenicity degrees. 

Therefore, the abundance of cathelicidins in milk samples is directly influenced by the type of pathogen that caused the mastitis, but it always remained high compared to controls, in which the presence of these proteins was not detected at all. 

In conclusion, because of this characteristic and their positive correlation with SCC, cathelicidins can represent a good marker for an early diagnosis of clinical or subclinical mastitis. Despite that, they cannot be used for treatment indications since they do not possess sufficient discriminatory power to indicate the causative pathogen.

#### 5.1.7. β-Lactoglobulin

β-lactoglobulin (BLG) is a major whey protein of the lipocalin family, accounting for approximately 53% of total whey proteins, and can bind to numerous hydrophobic molecules, suggesting a transport function [54,65,66]. BLG also exhibits iron-binding properties that may contribute to antimicrobial defense in milk [66]. Puppel et al. [65] reported pathogen-dependent modulation of BLG concentrations in mastitic milk: levels were increased in infections caused by *Staphylococci* spp. (>4.8 g/L vs. <3.2 g/L in controls), reduced in *Streptococcus* spp. mastitis (3.03 ± 0.29 g/L vs. 4.58 ± 0.21 g/L in controls), and unchanged in mastitis caused by *Enterobacteriaceae* spp. (3.83 ± 0.18 g/L in controls vs. 3.19 ± 0.14 g/L). These findings indicate that BLG responses vary according to the etiological agent. In addition, it has been reported that polymorphism in the BLG gene were associated with SCC [54]. Cows carrying the AA genotype showed significantly lower SCC compared with AB and BB genotypes, suggesting a greater tolerance to mastitis in this group. Overall, BLG appears to act as a pathogen-dependent component of the mammary immune response, potentially linked to its iron-binding capacity. Although these characteristics highlight BLG as a promising pathogen-specific indicator of udder health, further studies are needed to clarify its regulatory mechanisms and validate its diagnostic utility. 

#### 5.1.8. General Considerations on Protein Biomarkers for the Diagnosis of Mastitis

Taken together, the proteins reviewed in this section highlight the complexity of the mammary gland immune response and the potential of milk- and serum-derived molecules as biomarkers for mastitis. Although each reported protein shows a characteristic pattern of modulation in relation to the type, stage and severity of infection, none of them alone provides sufficient sensitivity and specificity to reliably discriminate all mastitis conditions. Their concentrations are influenced by multiple physiological factors (such as parity and stage of lactation, or days in milk), by the magnitude of the inflammatory response, and, most importantly, by the nature of the causative pathogen.

Nonetheless, several promising trends emerged. LTF, cathelicidins, and NAGase consistently increase during mastitis, particularly in more severe cases, reflecting local tissue inflammation. However, LTF, despite its strong correlation with SCC, is influenced by seasonality and stage of lactation. NAGase may vary according to days in milk. Cathelicidins may serve as useful early markers of both clinical and subclinical mastitis, but they do not possess sufficient discriminatory power to identify the causative pathogens. DEFB4 shows unique biphasic behavior, with an unexpected decrease in subclinical mastitis that may indicate early consumption of constitutive defensins, followed by a marked systemic and local upregulation during acute mastitis. Vitronectin and PON1 offer additional insights into systemic inflammatory and oxidative responses, but their diagnostic value remains limited: vitronectin shows only moderate specificity, and PON1 does not perform better than SCC alone. Finally, BLG shows pathogen-specific modulation and genetic associations that may help explain individual variability in udder health status.

Overall, the available evidence indicates that the combined evaluation of multiple proteins may improve the accuracy of mastitis detection, support the differentiation between clinical and subclinical infections, and offer pathogen-specific information. However, further research is required to harmonize analytical methods, define robust diagnostic thresholds, and validate biomarker combinations in larger field populations.

### 5.2. Glucidic Biomarkers for Mastitis

#### Lactose

Although fewer glucidic components have been proposed as mastitis indicators compared with protein or molecular markers, lactose has received attention due to its physiological role. Lactose is a disaccharide consisting of one glucose and one galactose molecule linked by a β-1,4-glycosidic bond. It is the main carbohydrate found only in mammalian milk and contributes to the maintenance of the blood–milk barrier by regulating the osmotic balance between blood and the alveolar lumen in the mammary gland [67]. It is the major milk solid in cows, in which about 20% of circulating glucose is converted into lactose during the lactation period [68].

Several studies have investigated variations in milk lactose levels in the presence of mammary gland inflammation, so in some studies this trend has also been investigated in mastitic milk samples. Antanaitis et al. [4] showed a reduction in lactose concentration in milk samples from cows with subclinical mastitis. This reduction was attributed to the impairment of lactose synthesis due to mammary gland tissue damage and the ability of certain pathogens to utilize lactose as a metabolic substrate [4,67]. Specifically, the lowest lactose content was observed in milk samples from cows infected with *S. agalactiae* and *S. aureus* (values ranging between 4.25% and 4.30%), while the highest lactose content was found in milk samples infected with serogroup G *Streptococci* (4.55 ± 0.12%) [4]. In agreement with these results, in an earlier study, a decrease in the lactose content was observed in samples derived from mastitic cows, but no significant differences related to the type of pathogen causing the disease were indicated [69].

This reduction in lactose content was relatively small, limiting the usefulness of lactose as a possible marker for the diagnosis of mastitis. For this reason, this parameter has been studied in association with SCC and NAGase activity, which not only highlights a greater responsiveness of the latter compared to lactose but also a significant negative correlation between lactose and SCC, as already previously stated [70].

In conclusion, although lactose concentration is routinely detected in dairy farms, its use as a unique biomarker for subclinical mastitis is limited, due to the small reduction observed in affected milk. However, when evaluated in combination with other parameters, particularly SCC, with which it shows a strong negative correlation, lactose may still provide useful information for mastitis detection.

### 5.3. Molecular Biomarkers for Mastitis

Recent research on mastitis diagnostics has increasingly focused on molecular indicators released during mammary gland damage and immune activation. Among these, damage-associated molecular patterns (DAMPs) such as mitochondrial DNA (mtDNA), and regulatory molecules like microRNAs (miRNAs) have gained attention for their potential to reflect early pathological processes that precede or accompany classical inflammatory changes. The following subsections present the main findings regarding mtDNA and miRNAs, highlighting their biological relevance, diagnostic potential, and the current limitations that still restrict their practical implementation.

#### 5.3.1. Mitochondrial DNA

During mastitis, one of the first phases of infection involves damage by pathogens to the epithelial cells of the mammary gland responsible for milk synthesis during lactation [71]. This damage can cause cell death, leading to a release into the extracellular environment of all the components normally contained in cells (DAMPs), including mitochondria, which release mtDNA following lysis. In physiological conditions, mtDNA release does not occur since the apoptosis process involves the phagocytosis of the apoptotic bodies containing the cellular components [46]. In a pilot study, Leishangthem et al. [46] investigated mtDNA release in the milk and serum of healthy cows compared to those affected by subclinical mastitis, given the association between mastitis and mammary epithelial cells destruction. They observed a significant increase in the mtDNA content in both the milk and serum of cows with subclinical mastitis compared to healthy samples. The cellular mtDNA content, indicated by the mitochondrial genome to nuclear genome ratio (mtDNA/nDNA), has been proposed as a biomarker for mitochondrial dysfunction in various human diseases, such as diabetes, cancer, and microbial infections [72], which suggested the quantification of extracellular mtDNA as a potential screening method and biomarker for diagnosing mastitis and assessing the tissue damage extent. In conclusion, mtDNA could be a good method to determine the extent of tissue damage, but further studies are necessary before proposing it as a biomarker as it could be less applicable due to its low specificity for mastitis, because tissue damage with the release of DAMPs can also be a consequence of other problems that can occur in the udder.

#### 5.3.2. MicroRNAs

MiRNAs are short, non-coding RNA molecules, typically 20–22 nucleotides in length, with a crucial role in the post-transcriptional regulation of gene expression [73,74]. Their precursors are synthesized in the nucleus and transported to the cytoplasm, where they are cleaved to form mature miRNA duplexes [75]. Mature miRNAs regulate gene expression by binding to the 3′ or 5′ untranslated regions (UTRs) of target mRNA, leading to its degradation or translational repression [42,75]. MiRNAs are actively released into body fluids like milk, where they remain remarkably stable and resistant to degradation from RNases, acidic conditions, and freeze–thaw cycles [76]. Since their expression levels change in response to pathological states such as infection, they are considered excellent candidates as biomarkers for animal health [75]. 

In the following section, the main miRNAs investigated as potential biomarkers of mastitis are presented according to their proposed functional classification: (I) miRNAs acting as general indicators of inflammation, showing non-specific upregulation (e.g., miR-21, miR-155); (II) miRNAs with potential value for pathogen discrimination, exhibiting distinct expression profiles depending on the etiological agent (e.g., miR-144, miR-451); (III) circulating miRNAs (c-miRNAs) detected in blood, which may serve as systemic indicators of mammary gland health.

(I) miRNAs as general markers of inflammation. A primary application of miRNA analysis in mastitis is the identification of general inflammatory markers in milk. Several studies have identified a core set of miRNAs that are consistently upregulated during mammary inflammation. For example, significantly increased concentrations of miR-21, miR-146a, miR-155, miR-222, and miR-383 were observed in mastitic milk, with their expression showing a positive correlation with the California Mastitis Test (CMT) score [76]. Many of these miRNAs are linked to pathways central to both inflammation and cancer. For instance, miR-21 is known to be overexpressed in inflammatory conditions and can be stimulated by lipopolysaccharides (LPS) [77], while miR-146a is a key regulator of the innate immune response, targeting TRAF6 and IRAK1 [78,79]. Similarly, another study identified a set of four miRNAs (miR-26a, miR-142-5p, miR-146a, and miR-223) that were overexpressed in cases of subclinical mastitis [80]. Their expression was positively correlated with both the CMT score and levels of immune mRNAs like IL1B and TNF-α. Notably, their levels were also influenced by the lactation stage. Receiver Operating Characteristic (ROC) curve analysis revealed that miR-142-5p, miR-146a, and miR-223 had high accuracy, supporting their potential for the early diagnosis of subclinical mastitis. However, the miRNA profile is not always consistent across studies. In contrast to the above findings, Srikok et al. [81] observed a significant decrease in the expression of miR-146a and miR-155 in mastitic skim milk samples, while miR-29b-2 and miR-184 were elevated. They highlighted miR-29b-2 as a particularly promising biomarker for mastitis due to its high specificity (81%), sensitivity (96%), and accuracy (89%). These conflicting reports underscore the sensitivity of miRNA profiling to factors such as pathogen type, infection stage, and sample matrix (e.g., whole vs. skim milk), necessitating standardized protocols for their validation as reliable biomarkers.

Among the miRNAs investigated as generale indicators of mammary inflammation, miR-223 and miR-26 emerged from our work [43] as markers with distinct expression patterns and diagnostic implications. Both were quantified in milk and in the culture medium of isolated immune cell subsets (lymphocytes, monocytes, and neutrophils), providing complementary evidence of their biological origin and functional relevance. Their behaviour in milk differed markedly. miR-223 was significantly overexpressed only in cows with acute mastitis, suggesting that it reflects an acute, high-grade inflammatory response rather than a general marker of minor or chronic inflammation. By contrast, miR-26 showed a consistent downregulation across all affected groups (acute, chronic and susceptible) relative to healthy controls, indicating a broader association with udder pathology that is independent of disease severity. 

**Figure 1:**
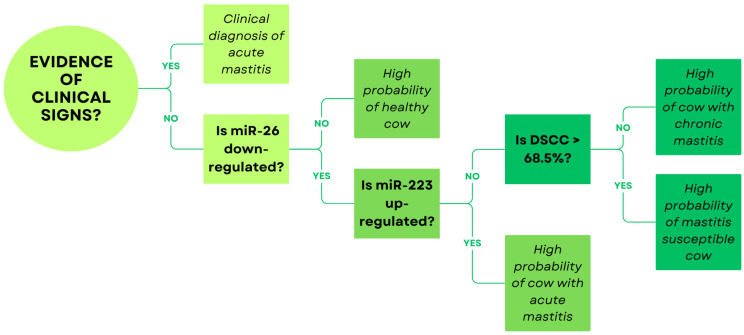
This flowchart illustrates a conceptual model of a stepwise approach that combines molecular and cytological markers to enhance the sensitivity and specificity of mastitis detection using non-invasive milk samples. The first step evaluates the presence of clinical signs: (1a) If clinical signs are present, the cow is classified as having acute mastitis; (1b) if absent, miR-26 expression is examined. (2a) Normal miR-26 levels indicate a high probability of a healthy cow, whereas (2b) downregulation of miR-26 requires assessment of miR-223. (3a) If miR-223 is not upregulated, the DSCC is evaluated; (3b) if miR-223 is upregulated, the cow is probably affected by acute mastitis. (4a) A DSCC below 68.5% suggests chronic mastitis, while (4b) values above this threshold indicate a cow with high susceptibility to mastitis. DSCC = differential somatic cell count.

Taken together, these findings indicate that miR-223 may be useful as an indicator of acute mammary inflammation, whereas miR-26 could serve as a more general marker of udder health status. In Figure 1, we proposed a conceptual model flowchart outlining a possible diagnostic strategy that integrates differential somatic cell count (DSCC) with the expression patterns of miR-223 and miR-26. DSCC is a refined indicator of udder inflammation, quantifying the proportion of immune cells (lymphocytes and neutrophils) on the total amount of somatic cells, with a threshold of 68.5% used to differentiate healthy from mastitic cows [32,82]. This approach is intended to support clinicians and researchers in achieving a more accurate interpretation of udder health status than that obtainable through SCC alone. By combining an immune system activation indicator (DSCC) with an acute phase-associated c-miRNA (miR-223) and a broadly responsive marker of mammary dysfunction (miR-26), the flowchart illustrates how multilevel biomarker assessment could improve the discrimination between healthy, subclinically inflamed, and acutely mastitic quarters. 

(II) miRNAs for pathogen discrimination. Beyond detecting general inflammation, specific miRNA signatures may help discriminate between the pathogens causing mastitis. In a study using an experimental infection model, Luoreng et al. [41] observed differential miRNAs expression in response to *S. aureus* versus *E. coli*. They noted a significant overexpression of miR-144 and miR-451 in milk from cows with *S. aureus*-induced mastitis, whereas the expression of these same miRNAs was decreased in samples from cows with *E. coli*-induced mastitis. These data suggests that the miR-144/miR-451 signature could serve as a potential biomarker for differentiating between these two major pathogens. In the same study, miR-7863 was found to be upregulated in response to both pathogens, positioning it as a more general biomarker for infection, in contrast to the discriminatory potential of the others.

(III) Circulating miRNAs as Systemic Indicators. Analyzing miRNAs in peripheral blood offers a less invasive method for monitoring the systemic effects of a local infection like mastitis. A study of c-miRNAs found that while the expression of miR-146a, miR-155, miR-222, and miR-383 did not change in the serum of mastitic cows, miR-21 was overexpressed [83]. This suggests that a local inflammatory process can indeed alter systemic c-miRNA profiles and that miR-21 could act as a blood-based indicator of mastitis. Furthermore, tracking miRNA expression over time can reveal biomarkers for early diagnosis. In fact, in a bovine mastitis model induced by *S. aureus*, Luoreng et al. [42] monitored the miRNA expression profile in peripheral blood over seven days. They identified significant changes on days five and seven, with an upregulation of miR-320a, miR-19a, and miR-19b, and a downregulation of miR-143, miR-205, and miR-24. More importantly, they found two miRNAs whose expressions changed consistently from the very first day of infection: miR-1301 was upregulated and miR-2284r was downregulated from day one through day seven. This early and sustained change suggests they could be effective peripheral blood biomarkers for the early diagnosis of *S. aureus*-induced mastitis.

#### 5.3.3. Concluding Remarks on Molecular Biomarkers

Overall, mtDNA and miRNAs represent emerging molecular biomarkers that provide valuable mechanistic insight into mastitis pathophysiology. mtDNA release reflects epithelial cell damage and may help estimate the extent of tissue injury, though its low specificity limits its diagnostic utility in the absence of supportive indicators. Conversely, miRNAs, summarized in Table 3, show a more potentially powerful diagnostic profile: some act as a general marker of inflammation, others display pathogen-specific expression patterns, and a subset of c-miRNAs may serve as systemic indicators of mammary health. As methodological standardization advances and multi-marker approaches combining miRNAs with established indicators (e.g., SCC, DSCC) are further validated, these molecular biomarkers may contribute significantly to more accurate and earlier mastitis diagnosis in dairy cattle.

## 6. Conclusions

SCC remains a reliable and widely used indicator of mammary gland inflammation, although its diagnostic performance may be influenced by physiological factors and it does not provide pathogen-specific information [34]. Recent advances in protein- and molecular-based biomarkers, particularly miRNAs, offer promising complementary tools for improving the early detection of clinical and subclinical mastitis, but none currently show sufficient robustness to be used as a stand-alone diagnostic method, and require technical skill and sophisticated infrastructure and facilities. Future progress in mastitis diagnosis will likely depend on the validation of integrated multi-biomarker panels and on the development of cost-effective, rapid, and farm-applicable diagnostic tools to translate scientific progress into practical benefits for the dairy industry.

## Figures and Tables

**Table 1:** Summary of potential biomarkers highlighted in the review and their main characteristics regarding the type of sample, mastitis, and their trend in case of mastitis. Expression indicates upregulation (Up) or downregulation (Down) compared to healthy controls. PR = protein biomarker; MO = molecular biomarker; GL = glucidic biomarker; SCM = subclinical mastitis; CM = clinical mastitis.

Sample	Marker	Mastitis	Molecule	Expression	Reference
**Milk and serum**	PR	SCM	**DEFB4**	Down	[44]
	PR	SCM	**PON1**	Down	[45]
	MO	SCM	**mtDNA**	Over	[46]
**Serum**	PR	SCM	**SAA**	Over	[47]
	PR	CM	**DEFB4**	Over	[44]
	PR	CM, SCM	**Vitronectin**	Over	[48]
**Milk**	PR	CM, SCM	**LTF**	Over	[49]
	PR	CM, SCM	**IgG**	Over	[50]
	PR	SCM	**Hp and AGP**	Over	[47]
	PR	SCM	**MAA**	Over	[51]
	PR	CM, SCM	**NAGase**	Over	[52]
	PR	CM, SCM	**Cathelicidins**	Over	[53]
	GL	SCM	**Lactose**	Down	[4]
	PR	CM, SCM	**BLG**	Down	[54]

**Table 2:** Lactoferrin (LTF) concentrations in milk from healthy and mastitic cows, as reported in different studies. Values are expressed in mg/mL. The table includes the method of detection and corresponding references.

Healthy (mg/mL)	Subclinical Mastitis (mg/mL)	Method of Detection	Reference
0.03 ± 0.01	0.1 ± 0.02	ELISA kit (Biopanda Reagents, Belfast, UK)	[50]
0.05 ± 0.01	0.07 ± 0.01	ELISA kit (Bethyl Laboratories Inc., Montgomery, TX, USA)	[55]
5.12 ± 1.77	5.45 ± 1.68 (major mastitis pathogens)	ELISA kit (Bethyl Laboratories Inc., Montgomery, TX, USA)	[49]
	5.95 ± 1.65 (minor mastitis pathogens)		
~0.25 ± 0.04	~0.84 ± 0.15	ELISA kit (Life Diagnostics, West Chester, PA, USA)	[47]

**Table 3:** Summary of potential miRNAs biomarkers for an early mastitis diagnosis highlighted in the review. Expression indicates upregulation (Up) or downregulation (Down) compared to healthy controls. CM = clinical mastitis; SCM = subclinical mastitis; EIM = experimentally induced mastitis.

Sample	Mastitis	MiRNA	Expression	Reference
**Milk**	CM, SCM	miR-21	Up	[76]
		miR-146a	Up	
		miR-155	Up	
		miR-222	Up	
		miR-383	Up	
**Serum**	CM, SCM	miR-21	Up	[83]
**Milk**	EIM (*E. coli*)	miR-144, miR-451	Down	[41]
	EIM (*S. aureus*)	miR-144, miR-451	Up	
	EIM (*E. coli*, *S. aureus*)	miR-7863	Up	
**Blood**	EIM (*S. aureus*)	miR-1301	Up	[42]
	EIM (*S. aureus*)	miR-2284r	Down	
**Skim milk**	CM, SCM	miR-29b-2	Up	[81]
		miR-184	Up	
		miR-146a	Down	
		miR-148a	Down	
		miR-155	Down	
**Milk**	SCM	miR-142-5p	Up	[80]
		miR-146a	Up	
		miR-223	Up	
**Milk**	CM, SCM	miR-26	Up	[43]
		miR-223	Up	

## Data Availability

No new data were created or analyzed in this study. Data sharing is not applicable to this article.

## Abbreviations

The following abbreviations are used in this manuscript:

aDCT	Antibiotic dry cow therapy
*S. aureus*	*Staphylococcus aureus*
*S. agalactiae*	*Streptococcus agalactiae*
*E. coli*	*Escherichia coli*
SCC	Somatic cell count
CMT	California mastitis test
miRNA	MicroRNA
LTF	Lactoferrin
IgG	Immunoglobulin G
Hp	Haptoglobin
AGP	α-1-acid glycoprotein
SAA	Serum amyloid A
MAA	Milk amyloid A
DEFB4	β-defensin 4
PMECs	Primary mammary epithelial cells
PON1	Paraoxonase 1
HDL	High-density lipoprotein
NAGase	N-acetyl-β-D-glucosaminidase
BLG	β-lactoglobulin
DAMPs	Damage-associated molecular patterns
mtDNA	Mitochondrial DNA
DSCC	Differential somatic cell count
CM	Clinical mastitis
SCM	Subclinical mastitis
EIM	Experimentally induced mastitis
